# The genus *Plateros* Bourgeois, 1879 (Coleoptera, Lycidae): Two new species from Central Vietnam

**DOI:** 10.3897/zookeys.1272.168039

**Published:** 2026-03-10

**Authors:** Sergey V. Kazantsev, Thai Hong Pham

**Affiliations:** 1 Insect Centre, 13–326 Donetskaya Str., Moscow 109651, Russia Vietnam Academy of Science and Technology Hanoi Vietnam; 2 Mientrung Institute for Scientific Research, Vietnam National Museum of Nature, VAST, 321 Huynh Thuc Khang, Hue, Vietnam Insect Centre Moscow Russia; 3 Graduate School of Science and Technology, Vietnam Academy of Science and Technology, 18 Hoang Quoc Viet, Hanoi, Vietnam Vietnam National Museum of Nature Hue Vietnam

**Keywords:** Bach Ma national park, distribution, Oriental region, taxonomy

## Abstract

Two new species of the genus *Plateros* Bourgeois, 1879, *P. bachmaensis***sp. nov**. and *P. phulocensis***sp. nov**., are described from Bach Ma National Park, central Vietnam. *Plateros bannaensis* Kazantsev, 2017 is recorded from Vietnam for the first time, found in Phia Oac-Phia Đen National Park, Cao Bang province, in northern Vietnam. This addition to the fauna of the region complements the recently published review of *Plateros* of Indochina, increasing the number of its species to 92, and the number of species registered in Vietnam, with the discovery of a species previously known only from Yunnan, to 73. A full list of *Plateros* species of Vietnam is provided.

## Introduction

The genus *Plateros* Bourgeois, 1879 is the largest in the family of net-winged beetles and includes over 900 species (Kleine 1933; Bocakova 2001; Kazantsev 2011). Most *Plateros* from Indochina were described by the French coleopterist Maurice Pic in the first half of the twentieth century (Pic 1916, 1921–1922, 1923, 1924–1939, 1927, 1939, 1942). However, following resumption of studies at the turn of the century, now based on the examination of aedeagal structures (Bocakova 1997; Kazantsev 1993, 2005, 2011, 2017, 2021; Kazantsev and Yang 1999), more than 20 synonymies and homonymies have been established, while the number of species of the genus known in the region has more than doubled.

The present study is a further contribution to the knowledge of *Plateros* of Vietnam. Examination recently collected material from Vietnam in the Naturkundemuseum, Erfurt has led to the discovery of two yet undescribed *Plateros* species and a species previously known only from China, which brings the number of Vietnamese species to 73 and the number reported from Indochina to 92. Description of the new species is given below, along with a list of all known species of *Plateros* of Vietnam.

## Material and methods

For examination the beetles were relaxed in water, then their detached abdomina were kept for up to 12 h in 10% KOH at room temperature. The KOH-treated aedeagi were then placed in microvials with glycerin for photographing. A MSP-1 zoom stereoscopic dissecting microscope with 8–80× magnification range was used. Photographs were taken with a Canon EOS 6D camera and a Canon MP-E 65 mm lens.

### Abbreviations used for the collections

**ICM** Insect Center, Moscow, Russia;

**NME** Naturkundemuseum, Erfurt, Erfurt, Germany;

**VNMN** Vietnam National Museum of Nature, Hanoi, Vietnam.

## Taxonomy

### Family Lycidae Laporte, 1838

**Subfamily Lycinae Laporte, 1838**

**Tribe Platerotini Kleine, 1928**

#### Plateros

Taxon classificationAnimaliaColeopteraLycidae

Bourgeois, 1879

205B6922-F7DC-5308-A876-D729FBC97D1F

Plateros Bourgeois, 1879: xix.
= Calleros Gorham, 1881 (synonymy by Bocakova 2001: 75).
= Calloplateros Pic, 1923 (synonymy by Bocakova 2001: 75).
= Cautiroides Pic, 1921 (synonymy by Bocakova 2001: 75).
= Costatoplateros Pic, 1949 (synonymy by Bocakova 2001: 75).
= Ditoneces Waterhouse, 1879 (synonymy by Bocakova 2001: 75).
= Graciloplateros Pic, 1921 (synonymy by Bocakova 2001: 75).
= Libnetomorphus Pic, 1921 (synonymy by Bocakova 2001: 75)
= Melampyrus Waterhouse, 1879 (synonymy by Bocakova 2001: 75).
= Microplateros Pic, 1921 (synonymy by Bocakova 2001: 75).
= Planeteros Gorham, 1883 (synonymy by Bocakova 2001: 75).
= Tolianus Pic, 1921 (synonymy by Bocakova 2001: 75).

##### Type species.

*Eros brasiliensis* Lucas, 1857 (subsequent designation by Zaragoza-Caballero 1999).

##### Distribution.

All biogeographic realms. However, in the Palaearctic region, only in its southeast and absent in the Greater Antilles, Madagascar, New Zealand, and Melanesia/Polynesia; just one species in Australia (Kleine 1933; Kazantsev 2011).

#### Plateros bachmaensis sp. nov.

Taxon classificationAnimaliaColeopteraLycidae

29F775AF-B0DB-563F-9580-5F7324592F9C

https://zoobank.org/3DDA801A-3EAD-41A2-A3C3-5644C8968C06

Figs 1A, 1B, 2D, 2E

##### Type material.

***Holotype*** • ♂ (NME): Central Vietnam, Hue city, Phu Loc, Bach Ma NP, top area, 1250–1400 m, 16°11'39"N, 107°51'12"E, LFF, 5–9.V.2019, A. Weigel leg.

##### Distribution.

Central Vietnam (Bach Ma National Park).

##### Description.

**Figure 1.**
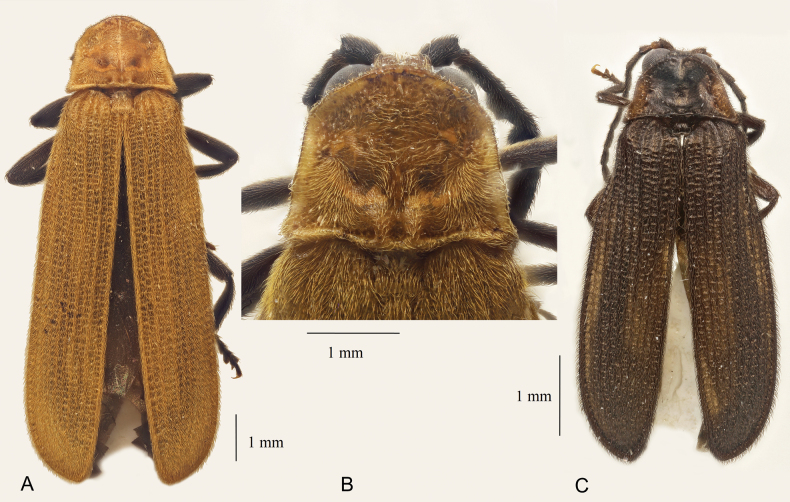
General view and details of *Plateros*, holotype males. **A, B**. *P. bachmaensis* sp. nov.; **C**. *P. phulocensis* sp. nov.; **A, C**. General view; **B**. Anterior part of body. Scale bars: 0.5 mm.

**Male**. Dark brown to black; head, pronotum, scutellum, elytra, front trochanters, and front femurs proximally orange testaceous (Fig. 1A, B).

Vertex with deep, round excavation between eyes. Interocular distance subequal in length to eye diameter. Labrum small, transverse, with conspicuous median incision anteriorly. Palps slender; ultimate palpomeres elongate, slightly widened distally, obliquely convex, and flattened at apex. Antennal sockets separated by minute lamina. Antennae reaching elytral 3/5, from antennomere 3 strongly dentate; antennomere 2 transverse, antennomere 3 ca 4.3 times longer than antennomere 2 and ca 1.1 times longer than antennomere 4, antennomeres 3–11 with relatively short decumbent pubescence (Fig. 1A, B).

Pronotum transverse, ca 1.3 times wider than long, trapezoidal, bisinuate basally, and moderately semicircularly produced anteriorly, with acute, slightly protruding laterally posterior and inconspicuous blunt anterior angles; medially with approximate posterior longitudinal ridges in posterior fifth and a pair of conspicuous round excavations in posterior third. Scutellum subquadrate, parallel-sided, almost truncate at apex (Fig. 1A, B).

Elytra moderately long, ca 3.3 times longer than wide at humeri, parallel-sided; interstices with even rows of regular subquadrate cells; pubescence dense, short, and decumbent (Fig. 1A).

Legs robust; femurs and tibiae straight, subequal in length.

**Figure 2.**
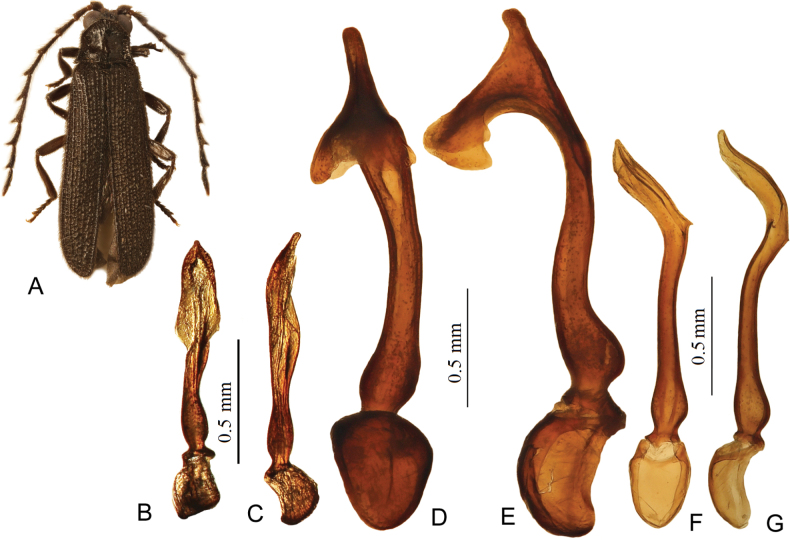
General view and details of *Plateros*, holotype males. **A–C**. *P. bannaensis*; **D, E**. *P. bachmaensis* sp. nov.; **F, G**. *P. phulocensis* sp. nov.; **A**. General view; **B, D, F**. Aedeagus, ventral view; **C, E, G**. Aedeagus lateral view. Scale bars: 0.5 mm. [**A**–**C**. After Kazantsev 2017].

Aedeagus large, ca 2.3 mm long, asymmetrical, with elongate, moderately curved, slightly widening before the apical portion median lobe; its apical portion dorsally produced with a pair of broad downward directed distal teeth; phallobase relatively broad, with incomplete median suture (Fig. 2D, E).

**Female**. Unknown.

***Length***: 10.2 mm. Width (humerally): 2.3 mm.

##### Etymology.

The name of the new species is derived from the name of the national park in Hue city in Central Vietnam, where the unique type specimen was collected.

##### Diagnosis.

*Plateros bachmaensis* sp. nov., habitually very similar to *P. macroimpressus* Kazantsev, 2021, is strikingly different in its aedeagus, with the apical portion of its median lobe dorsally produced and bearing a pair of broad downward directed distal teeth (Figs 1A, 1B, 2D, 2E).

#### Plateros phulocensis sp. nov.

Taxon classificationAnimaliaColeopteraLycidae

9AD76A59-96C3-5920-8994-EB4B0A375471

https://zoobank.org/2EC0B3C4-A62F-49A5-8F41-FD5616AAFC26

Figs 1C, 2F, 2G

##### Type material.

***Holotype*** • ♂ (NME): Central Vietnam, Hue city, Phu Loc, Bach Ma NP, top area, 1250–1400 m, 16°11'39"N, 107°51'12"E, LFF, 5–9.V.2019, A. Weigel leg. (NME); ***Paratypes*** • 18 ♂♂ (ICM, NME and VNMN), same data as holotype.

##### Distribution.

Central Vietnam (Bach Ma National Park).

##### Description.

**Male**. Dark brown to black; antennomere 2 distally and pronotum laterally light brown (Fig. 1C).

Vertex with deep, round excavation between eyes. Eyes large, interocular distance ca 1.4 times shorter than eye diameter. Labrum small, transverse, slightly concave anteriorly. Palps slender; ultimate palpomeres elongate, parallel-sided, obliquely truncate, and flattened at apex. Antennal sockets separated by minute lamina. Antennae reaching elytral middle, antennomeres 3–10 conspicuously flattened, but parallel-sided; antennomere 3 ca 1.5 times longer than antennomere 2 and ca 1.7 times shorter than antennomere 4; antennomeres 3–11 with relatively short erect pubescence (Fig. 1C).

Pronotum strongly transverse, ca 2 times wider than long, trapezoidal, slightly concave basally, and noticeably semicircularly produced anteriorly, with prominent, acute, protruding latero-posteriorly posterior and rounded anterior angles. Scutellum transverse, narrowing distally, slightly medially incised at apex (Fig. 1C).

Elytra elongate, broad, ca 2.7 times longer than wide at humeri, noticeably widening posteriorly, broadest at distal third; with four almost equally developed primary costae, only humeral costa considerably stouter in proximal third; interstices with even rows of irregular subquadrate cells; pubescence dense, short, and semi-erect (Fig. 1C).

Legs robust; femurs and tibiae narrow, subequal in length.

Aedeagus relatively small, ca 1.4 mm long, asymmetrical, with elongate, straight in proximal two thirds of median lobe; its apical third bent and bearing a pair of minute teeth at the base of the bend; phallobase broad, without sutures (Fig. 2F, G).

**Female**. Unknown.

***Length***: 5.0–6.4 mm. Width (humerally): 1.4–1.7 mm.

##### Etymology.

The name of the new species is derived from the name of the locality in Central Vietnam where the type series was collected.

##### Diagnosis.

*Plateros phulocensis* sp. nov. is similar to *P. tamdaoensis* Kazantsev, 2021, readily separable by the larger eyes, their diameter being ca 1.4 times greater than the interocular distance (ca 1.1 times smaller in *P. tamdaoensis*), considerably shorter antennomere 3, which is only 1.5 times longer than antennomere 2 (antennomere 3 ca 2.6 times longer than antennomere 2 in *P. tamdaoensis*), more produced posterior pronotal angles and noticeably widened posteriorly elytra (parallel-sided in *P. tamdaoensis*), as well as by the less pointed apically median lobe of the aedeagus (Figs 1C, 2F, 2G).

#### Plateros bannaensis

Taxon classificationAnimaliaColeopteraLycidae

Kazantsev, 2017

02479CD0-4E63-5857-84CD-DDBD2B34E88C

Fig. 2A–C

Plateros bannaensis Kazantsev, 2017: 243.

##### Type material.

***Holotype*** • ♂: China, S Yunnan, Xishuangbanna, 20 km NW of Jinghong, Man Dian NNNR-office, 22°07.80'N, 100°40.05'E, 740 m, LFF, 24.V.2008, A. Weigel leg. (NME); ***Paratypes*** • 15 ♂♂ and 2 ♀♀, same label (ICM and NME).

##### Additional material.

Vietnam: • ♂, N Vietnam, Cao Bang Prov., vic. Tinh Tuc, Son Dong, Nui Pia Oac Nature Reserve, 850–1300 m, 22°37'55"N, 105°52'98"E, light trap, 9–15.V.2014, A. Weigel leg. (NME).

##### Distribution.

Southern Yunnan (Xishuangbanna); northern Vietnam (Nui Pia Oac National Park). First record for Vietnam.

##### Remark.

In the recently published review of the genus *Plateros* of Indochina (Kazantsev 2021) certain errors have been discovered. The captions to figures 29–40 (page 52): “29 — *P. chinensis*; 30 — *P. nonus*; 31 — *P. cinis*’ should read: ’29 — *P. igneus*; 30 — *P. nanensis*; 31 — *P. nemo*”. Also, on page 67, left column, in the *Plateros propinquus* (Waterhouse, 1879) bloc instead of “*Ditineces chinensis* Waterhouse, 1879: 32" there should be “*Ditoneces propinquus* Waterhouse, 1879: 32".

### A checklist of *Plateros* species of Vietnam

1. *Plateros alitecostatus* Kazantsev, 2011: 189. Northern Vietnam (“Tonkin”).

= *Plateros diversecostatus* Pic, 1942: 6 (homonymy by Kazantsev 2011: 189).

2. *Plateros amplipennis* Pic, 1921: 7. Southern Vietnam: “Saigon”.

3. *Plateros ater* Pic, 1931: 97. Northern Vietnam (Hoa Binh).

4. *Plateros bachmaensis* sp. nov. Central Vietnam (Bach Ma National Park).

5. *Plateros bannaensis* Kazantsev, 2017: 243. Northern Vietnam (Nui Pia Oac National Park); southwestern Yunnan (Xishuangbanna). First record for Vietnam.

6. *Plateros baolokensis* Kazantsev, 2021: 53. Southern Vietnam (Bao Lak, 1500 m); Laos.

7. *Plateros basipes* Pic, 1942: 5 (*Ditoneces*). Northern Vietnam (“Tonkin”).

8. *Plateros bellipratensis* Kazantsev, 2021: 53. Northern Vietnam (Cuc Phuong National Park, 320 m).

9. *Plateros belokobylskyi* Kazantsev, 2011: 168. Northern Vietnam (Cuc Phuong National Park, 200–300 m).

10. *Plateros bifoveiceps* Pic, 1921: 4 (*Ditoneces*). Vietnam (Na Hang, Hoa-Binh, Gialai-Contum); Laos, Thailand, Cambodia, Malaysia, and Singapore. Widespread and common.

11. *Plateros binhanus* Pic, 1925: 10 (*Ditoneces*). Vietnam (Hoa Binh, Gialai-Kontum); Laos and Thailand.

12. *Plateros brevehumeralis* Pic, 1927: 35. Northern Vietnam (“Tonkin”).

13. *Plateros chapaensis* Pic, 1923: 11 (*Ditoneces*). Northern Vietnam (Chapa, 1600–2000 m).

14. *Plateros chinensis* Waterhouse, 1879: 29. Vietnam (Tam Dao, Gialai-Contum); Thailand, Cambodia, and China.

= *Plateros annamitus* Pic, 1921: 7 (synonymy by Kazantsev 2021: 55).

= *Plateros elisus* Pic, 1921: 7 (synonymy by Kazantsev 2021: 55).

= *Plateros flavomarginatus* Kleine, 1936: 264 (synonymy by Bocakova 1997: 177).

= *Plateros formosanus* Pic, 1921: 7 (synonymy by Bocakova 1997: 177).

= *Plateros sycophanta* Fairmaire, 1888: 352 (synonymy by Bocakova 1997: 177).

15. *Plateros ciceroi* Kazantsev, 2011: 189. Northern Vietnam (‘Tonkin’).

= *Ditoneces tonkineus* Pic, 1931: 97 (homonymy by Kazantsev 2011: 189).

= *Ditoneces tonkineus* var. *discicollis* Pic, 1942: 6 (synonymy by Kazantsev 2021: 57).

16. *Plateros cinis* Kazantsev, 2011: 168. Northern Vietnam (Cuc Phuong National Park, 200 m).

17. *Plateros cochinensis* Kazantsev, 2011: 168. Southern Vietnam (Gialai-Contum).

18. *Plateros deinceps* Kazantsev, 2011: 169. Northern Vietnam (Hoa Binh, 1100–1200 m).

19. *Plateros depressicornis* Pic, 1942: 6. Northern Vietnam (‘Tonkin’).

20. *Plateros disconiger* Pic, 1926: 32 (*Ditoneces*). Northern Vietnam (‘Tonkin’).

21. *Plateros donckieri* Pic, 1923: 53 (*Ditoneces*). Northern Vietnam (Hoa-Binh, Cuc Phuong National Park).

22. *Plateros dulcis* Kazantsev, 2011: 169. Northern Vietnam (Hoa Binh).

23. *Plateros elongatissimus* Pic, 1923. Northern Vietnam (Hoa Binh).

= *Plateros elongatissimus* var. *bicolorithorax* Pic, 1926: 24 (synonymy by Kazantsev 2021: 57).

24. *Plateros faber* Kazantsev, 2011: 169. Northern Vietnam (Chapa, 1600 m, Rai Yen Tu Natural Reserve, 200 m, Nui Pia Oac Natural Reserve, 850–1300 m).

25. *Plateros fedorenkoi* Kazantsev, 2011: 169. Southern Vietnam (Lam Dong, 1400–1600 m).

26. *Plateros gavryushini* Kazantsev, 2017: 243. Northern Vietnam (Cuc Phuong National Park, 270 m), Thailand.

27. *Plateros gemellus* Kazantsev, 2021: 58. Northern Vietnam (Na Hang, Hoa Binh, Cuc Phuong National Park).

28. *Plateros gerstmeieri* Kazantsev, 2021: 58. Northern Vietnam (Cuc Phuong National Park, 160 m).

29. *Plateros gurkha* (Kazantsev, 2001): 14 (*Melaneros*). Northern Vietnam: Hoa Binh; northern Thailand: Chiang Mai; Nepal: Birganj, Karnali; India: Uttar Pradesh; southwestern China: Yunnan; and Cambodia.

= *Plateros anguliplanatus*Kazantsev 2021: 51 (synonymy by Kazantsev 2025b: 134)

30. *Plateros hoabinhensis* Kazantsev, 2011: 169. Northern Vietnam (‘Tonkin’).

= *Plateros binhanus* Pic, 1925: 10 (homonymy by Kazantsev 2011: 169).

31. *Plateros hoi* Kazantsev, 2005: 250. “Vietnam”.

32. *Plateros impressicollis* Pic, 1942: 5 (*Ditoneces*). Northern Vietnam (“Tonkin”).

33. *Plateros integer* Kazantsev, 2011: 171. Northern Vietnam (Hoa Binh, 1100–1200 m, Nui Pia Oac Natural Reserve, 850–1300 m).

34. *Plateros kabakovi* Kazantsev, 2011: 171. Northern Vietnam (Chapa, 1600–2000 m)

35. *Plateros kabakovianus* Kazantsev, 2017: 244. Northern Vietnam (Thai Nguen, 380 m).

36. *Plateros lacosus* Pic, 1926: 24. Northern Vietnam (“Tonkin”).

37. *Plateros laocaensis* Kazantsev, 2011: 172. Northern Vietnam: Chapa, 1950–2100 m, Cuc Phuong National Park, 380 m).

38. *Plateros laticornis* Pic, 1916: 16 (*Ditoneces*). Northeastern Thailand (Chiang Mai, Loei); S Vietnam and Laos.

39. *Plateros limbatus* Pic, 1926: 31 (*Ditoneces*). Northern Vietnam (“Tonkin”).

40. *Plateros loeiensis* Kazantsev, 2011: 172. Northern Vietnam (Tam Dao, 200 m); Thailand.

41. *Plateros magnicauda*Kazantsev 2021: 63. Northern Vietnam (Cuc Phuong National Park, 270–390 m).

42. *Plateros medvedevi* Kazantsev, 2017: 247. Southern Vietnam (Gialai-Kontum, 740 m).

43. *Plateros multiimpressus* Pic, 1926: 33. Northern Vietnam (Hoa Binh, Cuc Phuong National Park),; Thailand and Laos.

44. *Plateros napolovi* Kazantsev, 2005: 239. Northern Vietnam (Cuc Phuong, Thai Nguen).

45. *Plateros nitidus* Pic, 1938: 160. Northern Vietnam (Hoa Binh).

46. *Plateros nonus* Kazantsev, 2011: 174. Northern Vietnam (Cuc Phuong National Park, 200–300 m, Hoa Binh, 1100–1200 m); Laos.

47. *Plateros nox* Kazantsev, 2005: 244. Northern Vietnam (“Tonkin”).

= *Ditoneces atripennis* Pic, 1926: 32 (homonymy by Kazantsev 2005: 244).

48. *Plateros obscurior* Pic, 1938: 160. Northern Vietnam (‘Tonkin’).

49. *Plateros olexai* Kazantsev, 2017: 249. Northern (Tamdao, 900 m) and central Vietnam (Bach Ma National Park, 1250–1400 m).

50. *Plateros orlovi* Kazantsev, 2011: 175. Northern Vietnam (Chapa, 1900–2500 m).

51. *Plateros phulocensis* sp. nov. Central Vietnam (Bach Ma National Park).

52. *Plateros phungi* Pic, 1923: 58 (*Ditoneces*). Northern Vietnam (Son La); Laos.

53. *Plateros planatus* Waterhouse, 1879: 27. Vietnam; Laos, Thailand, Japan, Korea, China, and the Himalayas (northern India and Nepal).

= *Plateros fulgens* Kleine, 1933: 20 (synonymy by Bocakova 1997: 178).

= *Ditoneces hoanus* Pic, 1926: 32 (synonymy by Kazantsev 2011: 189).

= *Ditoneces incisicollis* Pic, 1921: 5 (synonymy by Bocakova 1997: 178).

= *Plateros koreanus* Kleine, 1936: 263 (synonymy by Kazantsev and Yang 1999).

= *Ditoneces pallidus* Pic, 1921: 5 (synonymy by Kazantsev 2011: 189).

= *Plateros purus* Kleine, 1926: 99 (synonymy by Kazantsev 2005: 243).

= *Ditoneces sulcatithorax* Pic, 1925: 18 (synonymy by Bocakova 1997: 178).

= *Plateros tuberculatus* Pic, 1921: 6 (synonymy by Bocakova 1997: 178).

54. *Plateros planatomimus*Kazantsev 2021: 66. Southern Vietnam (Nam Cat Tien National Park); Thailand and Laos.

55. *Plateros prolongatus* Pic, 1939: 31. Northern Vietnam (“Tonkin”).

56. *Plateros propinquus* Waterhouse, 1879: 32 (*Ditoneces*). Northern Vietnam (Tam Dao, 200 m, Thai Nguen, 300 m, Cuc Phuong); China.

57. *Plateros proplanatus* Kazantsev, 2021: 67. Central Vietnam (Dong Hoi, 200 m).

58. *Plateros prosvirovi* Kazantsev, 2017: 250. Northern Vietnam (Lao Kay, 1370–1440 m).

59. *Plateros pulverulentus* Kazantsev, 2011: 175. Northern Vietnam (Tamdao).

60. *Plateros purpureus* Pic, 1942: 6. Northern Vietnam (“Tonkin”).

61. *Plateros raotensis* Kazantsev, 2021: 67. Northern (Thai Nguen, 300 m) and central Vietnam (Dong Hoi, 600 m).

62. *Plateros reductetestaceus* Pic, 1938: 160. Northern Vietnam (“Tonkin”).

63. *Plateros robustithorax* Pic, 1923: 14. Northern Vietnam (Chapa).

64. *Plateros sarmentosus* Kazantsev, 2021: 69. Northern Vietnam (Cuc Phuong).

65. *Plateros semimarginatus* Pic, 1939: 31. Northern Vietnam (“Tonkin”).

66. *Plateros siniaevi* Kazantsev, 2021: 69. Southern Vietnam (Bao Lok, 1500 m).

67. *Plateros subductor* Kazantsev, 2011: 190. Northern Vietnam (“Tonkin”).

68. *Plateros subplanatus* Kazantsev, 2011: 175. Northern Vietnam (Chapa, Huang Lien Son Nature Reserve, 1600–2070 m).

69. *Plateros subvittatus* Pic, 1931: 97 (*Ditoneces*). Northern Vietnam (“Tonkin”).

70. *Plateros tamdaoensis* Kazantsev, 2021: 71. Northern Vietnam (Tam Dao, 900 m).

= *Plateros dinghuensis* Fang, Yang, Yang et Liu, 2024: 146 (synonymy by Kazantsev 2025a: 3).

71. *Plateros tenebrosus* Kazantsev, 2011: 175. Southern Vietnam (Nam Cat Tien National Park).

72. *Plateros tonkineus* Pic, 1926: 31. Northern Vietnam (“Tonkin”).

73. *Plateros xalinhensis* Kazantsev, 2021: 72. Northern Vietnam (Hoa Binh, 1120 m, Nui Pia Oac Natural Reserve, 850–1300 m).

## Discussion

The *Plateros* fauna of Vietnam is conspicuously more species-rich than that of any neighbouring country, even with comparable territory and sometimes as diverse as Vietnam in terms of general biodiversity, such as Thailand. For example, while the number of *Plateros* species in Vietnam is 73, there are only 21 registered members of this genus in Thailand, and only six in Cambodia (Kazantsev 2021). It is true, 25 of the valid *Plateros* names from Vietnam are authored by Maurice Pic (plus five replaced homonymous names), which sets the share of actually Pic’s taxa in the regional *Plateros* fauna at 41%. Only eight of these taxa have been identified and illustrated (Kazantsev 2021), 22 are still known only by their rather vague descriptions. For this reason, it is possible that some of the later described taxa may eventually turn out to be conspecific with Pic’s ones. Nevertheless, even if all of these 22 taxa prove to be senior synonyms, which is unlikely, the remaining 51 Vietnamese *Plateros* species will still surpass the number of Thai ones by more than 2.4 times.

One of the reasons that might explain this is that Vietnam seems to have been better explored, from Pic’s time, i.e. from the beginning of the 20^th^ century. Another reason could be that Vietnam’s rugged forested mountains have better preserved its beetle fauna than perhaps the more cultivated, less rugged landscapes in Thailand. And the third reason could be that Vietnam’s damp maritime climate just better suits the primary moist-forest-dwelling net-winged beetles than most of the biotopes found in Thailand. In any case, the exploration of the net-winged beetle fauna in the whole of Indochina needs to be continued, preferably on a wider scale.

## Supplementary Material

XML Treatment for Plateros

XML Treatment for Plateros bachmaensis

XML Treatment for Plateros phulocensis

XML Treatment for Plateros bannaensis

## Additional information

### Use of AI

No use of AI was reported.

### Author ORCIDs

Sergey V. Kazantsev https://orcid.org/0000-0001-6982-3337

Thai Hong Pham https://orcid.org/0000-0002-4763-3679

### Data availability

All of the data that support the findings of this study are available in the main text.
